# Novel Fe(II)-Based Supramolecular Film Prepared by Interfacial Self-Assembly of an Asymmetric Polypyridine Ligand and Its Electrochromic Performance

**DOI:** 10.3390/molecules30061376

**Published:** 2025-03-19

**Authors:** Xiya Chen, Xiaomeng Sun, Tingting Dai, Hongwei Wang, Qian Zhao, Chunxia Yang, Xianchao Du, Xiaojing Xing, Xinfeng Cheng, Dongfang Qiu

**Affiliations:** 1 College of Chemistry, Zhengzhou University, No. 100 of Kexue Road, Zhengzhou 450001, China; 2 College of Chemistry and Pharmaceutical Engineering, Nanyang Normal University, Nanyang 473061, China

**Keywords:** asymmetric polypyridine ligand, liquid–liquid interface self-assembly, supramolecular polymer, electrochromism

## Abstract

An asymmetric two-arm polypyridine ligand 4′-{4-[4-(2,2′-dipyridyl)phenyl]}-2,2′:6′,2′-terpyridine (**TPY-Ph-BPY**) with double coordination units was synthesized using the one-step Suzuki reaction. The metallic supramolecular film was subsequently obtained by the Fe^2+^-induced self-assembly method at the CHCl_3_-H_2_O interface, which displayed a distinct flat and continuous morphology. The supramolecular film-coated ITO electrode demonstrated a reversible electrochemical redox behavior with pronounced color changes between purple and light green. Its solid-state electrochromic device had an optical contrast (Δ*T*%) of 26.2% at λ_max_ = 573 nm with balanced coloring (*t*_c_ = 2.4 s) and bleaching (*t*_b_ = 2.6 s) times and a high current efficiency of 507.8 cm^2^/C. Moreover, good cycling stability with a long-term reversible color change was observed beyond 900 cycles. These results suggested the promising potential of the **TPY-Ph-BPY-Fe(II)** supramolecular film for electrochromic applications.

## 1. Introduction

Electrochromic (EC) materials [1,2] are extensively utilized in smart windows [3,4,5,6], next-generation displays [7,8,9], and wearable electronics [10,11] because of their simple and adjustable structure, low power consumption, flexibility, and stretchability [12]. Electrochromic materials are commonly found in metal oxides, small organic molecules, π-conjugated polymers, and metallic supramolecular polymers [13,14,15]. Among them, metallic supramolecular polymers have been widely investigated due to their combined properties of organic and inorganic electrochromic materials, showing better stability, diverse color changes, fast switching time, and other properties [16].

**Scheme 1:**
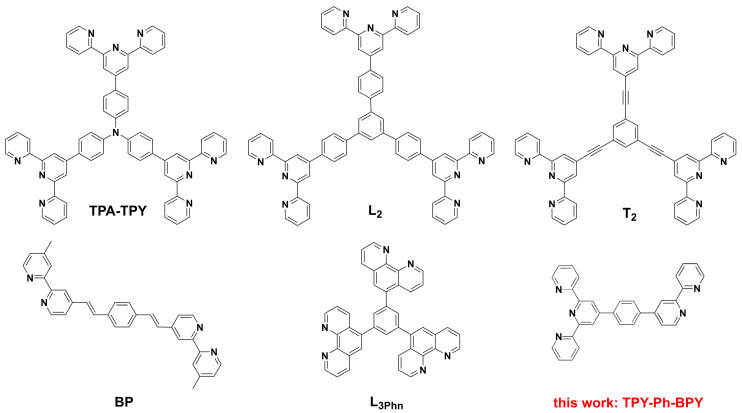
Chemical structures of the reported TPYs- and BPYs-based ligands, and the asymmetric polypyridine ligand **TPY-Ph-BPY** in this study.

Recently, metal ion-induced self-assembly of organic ligands at the two-phase interface [17] has been developed to form two-dimensional (2D) supramolecular EC films. This method has several advantages over traditional electropolymerization [18] and spin-coating [19] methods, such as: (1) mild operation conditions (room temperature and pressure), (2) simple and controllable parameters (concentration of a two-phase solution, resting time), (3) it is convenient to prepare the large-sized device by using a large-diameter container, (4) integrating the online synthesis and ordered assembly techniques effectively avoids the preprocessing or postprocessing problems, and (5) the obtained supramolecular layer is insoluble in traditional solvents, which is conducive to improving the cycle stability of the device. Three-arm terpyridine derivatives (3TPYs), such as TPA-TPY [20], L_2_ [21], and T_2_ [22], and a bipyridine derivatives (BPYs)-based L_3Phn_ [23] ligand as well as a two-arm BPYs (2BPYs)-based BP [24] ligand (Scheme 1) have been reported. From the topological analysis of the 3TPYs–Fe(II) supramolecular film, the branched organic cores in the 3TPYs ligands act as the three-nodes and the complex [Fe(TPY)_2_]^2+^ units formed by Fe^2+^-induced chelation of the TPY units from two adjacent ligand molecules serve as the two-nodes to expand its 2D spatial structure. However, in the 2BPYs–Fe(II) supramolecular polymer, the coordinated [Fe(BPY)_3_]^2+^ units serve as the three-nodes, while 1, 4-substituted phenyl groups act as the two-node effect.

Herein, an asymmetric polypyridine ligand 4′-{4-[4-(2,2′-dipyridyl)phenyl]}-2,2′:6′,2′-terpyridine (**TPY-Ph-BPY**) with two different coordination units (TPY and BPY) was designed to integrate the two-node ([Fe(TPY)_2_]^2+^ units) and three-node ([Fe(BPY)_3_]^2+^ units) effects (Scheme 1). The Fe^2+^-induced liquid–liquid interfacial self-assembly behavior and the EC performance of the formed **TPY-Ph-BPY-Fe(II)** supramolecular layer in the solid-state device were studied in detail. It was expected that both [Fe(BPY)_3_]^2+^ and [Fe(TPY)_2_]^2+^ coordination units were present in this metallic supramolecular film and a combination of their optoelectronic properties would achieve better electrochromic properties.

## 2. Results and Discussion

### 2.1. Synthesis of the **TPY-Ph-BPY** Ligand

As shown in Scheme S1, the one-step Suzuki reaction between phenylboronic acid-functionalized TPY and bromo-functionalized BPY was employed using the [Pd(PPh_3_)_2_Cl_2_] catalyst to synthesize the **TPY-Ph-BPY** ligand. The ^1^H NMR spectrum (Figure S1) indicates that an asymmetrical product has been obtained, in which the singlet 2H peaks at δ = 8.83 ppm correspond to the two hydrogen atoms at the 3′- and 5′-positions of the TPY unit, and the doublet 2H peaks at δ = 8.71, 8.52 and 7.65 ppm are, respectively, consistent with the two hydrogen atoms at the 6- and 6′′-positions of the TPY unit and the two hydrogen atoms at the 6- and 6′-positions of the BPY unit. The proposed structure was also confirmed by 13C NMR (Figure S2) and MALDI-TOF MS. The resulting ligand is soluble in CHCl_3_ and CH_2_Cl_2_ but difficult to dissolve in other solvents.

### 2.2. Continuous Titration Analysis

**Figure 1:**
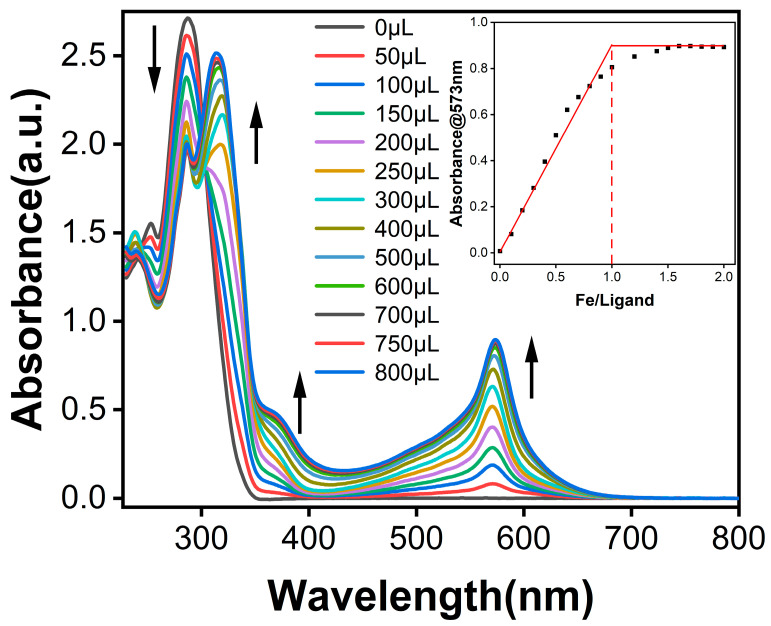
UV–vis spectroscopy of the 5 × 10^−5^ M **TPY-Ph-BPY** acetonitrile solution upon titration of 5 × 10^−3^ M Fe(BF_4_)_2_·6H_2_O aqueous solution (inset: absorbance change vs. the concentration ratio of Fe^2+^ to ligand at λ_max,abs_ = 573 nm).

As depicted in Figure 1, the asymmetric **TPY-Ph-BPY** ligand displayed a characteristic sharp absorption band at λ_max,abs_ = 287 nm, which originated from the singlet π→π* intraligand (^1^IL) transitions of both TPY and BPY units [25]. With the continuous addition of aqueous Fe^2+^ solution, the ^1^IL transition band was decreased gradually, while three new absorption peaks located at λ_max,abs_ = 320, 372, and 573 nm emerged and were intensified. These new absorption bands were, respectively, assigned to the ^1^IL, singlet ligand-to-ligand charge transfer (^1^LLCT) [26], and the spin-allowed dπ(M)-π*(TPY and BPY) metal-to-ligand charge transfer (^1^MLCT) transitions. Notably, compared with the ^1^MLCT bands of the reported Fe^2+^-TPYs (Fe^2+^-TPATPY, λ_max,abs_ = 580 nm [20]; Fe^2+^-L_2_, λ_max,abs_ = 580 nm [21]; Fe^2+^-T_2_, λ_max,abs_ = 588 nm [22]) and Fe^2+^-BPYs (Fe^2+^-BP, λ_max,abs_ = 568 nm [24]; Fe^2+^-L_3Phn_, λ_max,abs_ = 518 nm [23]) supramolecular systems, the broad and blue-shifted ^1^MLCT band was observed for this asymmetric ligand, suggesting that both the TPY and BPY units of the **TPY-Ph-BPY** ligand are involved in coordination process. Moreover, when the concentration ratio of Fe^2+^ to ligand exceeded 1.5 times, the ^1^MLCT absorption band intensity at λ_max,abs_ = 573 nm, reached its maximum (as shown in the inset of Figure 1). The inflection point value calculated by the double tangent method is about 1.0, which is extremely close to the ideal value for forming a perfect coordination polymer. These results confirm that the supramolecular polymer can be easily formed by Fe^2+^-induced self-assembly of the asymmetric **TPY-Ph-BPY** ligand at room temperature.

### 2.3. Preparation and Characterization of the **TPY-Ph-BPY-Fe(II)** Film

**Figure 2:**
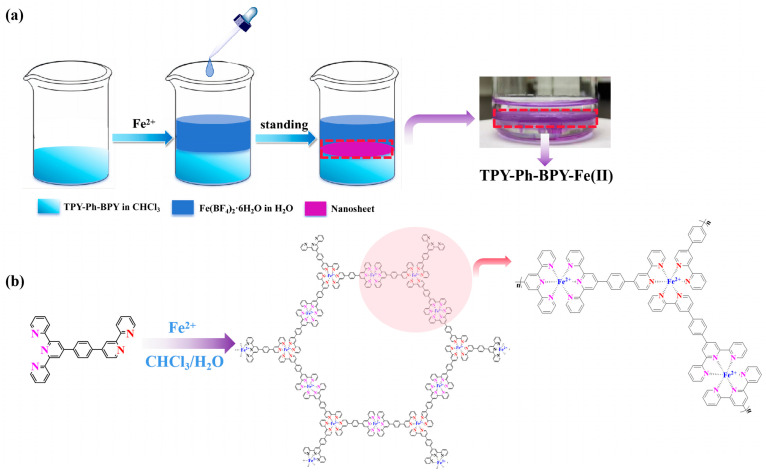
Interfacial assembly process (**a**) and proposal 2D structure (**b**) of the **TPY-Ph-BPY-Fe(II)** film.

On the basis of the homogeneous phase titration results, the formation of the supramolecular polymer at the CHCl_3_-H_2_O interface was tested at room temperature. The preparation process was described in the Experimental section and illustrated in Figure 2a. Keeping the concentration (10^−2^ M) of the Fe^2+^ aqueous solution and the concentration (10^−3^ M) of the ligand unchanged, a series of purple films with various thicknesses and growth qualities was obtained by adjusting the standing time from 3 h to 12 h. As shown in Figure S3a, the longer the standing time, the deeper the purple color and the thicker the film thickness. The film generally exhibits a flat and continuous morphological feature (Figure S3b), although cracks may appear for the thicker film due to the rigidity of the ligand structure. The average film thickness increases with the standing time, ranging from 98.32 nm to 3.26 μm (Figure S3c). These results indicate that the **TPY-Ph-BPY-Fe(II)** supramolecular polymer (Figure 2b) can also be formed in this heterogeneous system, and the two-dimensional (2D) film formed at the interface is easier to transfer to silicon wafer or ITO glass, which is beneficial for its structure characterization and optoelectronic property studies.

To explore the relationship between the morphology of the supramolecular film and the photoelectric properties, their UV–vis absorption spectra and cyclic voltammograms were monitored. As shown in Figure S4a, the UV–vis absorption spectra of the film-coated ITO glasses are similar to those in the continuous titration process. The characteristic ^1^IL, ^1^LLCT, and ^1^MLCT absorption bands at λ_max_ = 320, 372, and 579 nm intensified with the film thickness increase. However, when plotting the absorption intensity at λ_max_ = 579 nm against the standing time of the film formation (Figure S4b), it was found that the relationship between the increase in film thickness and the standing time is not entirely linear.

**Figure 3:**
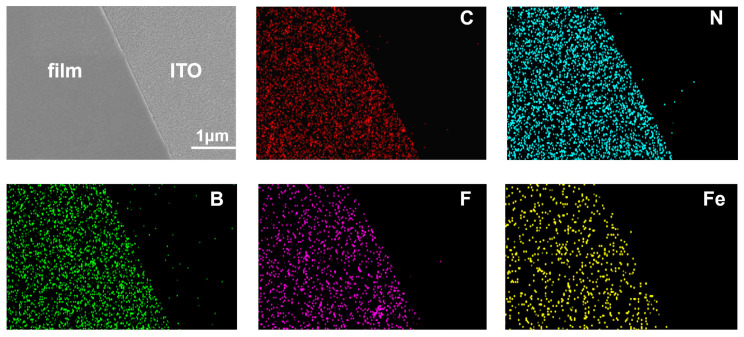
SEM-EDS mapping images of the **TPY-Ph-BPY-Fe(II)** film.

From the FT-IR spectra analyses (Figure S5), the characteristic peaks at 484 cm^−1^ and 738 cm^−1^ come from the benzene [27] and pyridine [28] rings, respectively. The characteristic C-N and C=C stretching vibration bands of the **TPY-Ph-BPY** ligand appear at 1467 cm^−1^ and 1583 cm^−1^ [25], which shifted to 1473 cm^−1^ and 1606 cm^−1^ for the coordinated **TPY-Ph-BPY-Fe(II)** film. Additionally, there exists a distinct stretching vibration at 1076 cm^−1^ [29], which corresponds to the BF_4_^−^ counter-anion in the **TPY-Ph-BPY-Fe(II)** film. Meanwhile, the energy dispersive spectroscopy (EDS) results show that the film has a uniform elemental distribution, mainly containing elements such as C, N, B, F, and Fe (Figure 3). The elemental ratio of N to Fe was determined to be 6.02:1 (Table S1), which is close to the theoretical ratio of 6:1 [24] in the generated [Fe(TPY)_2_]^2+^ and ([Fe(BPY)_3_]^2+^ units with hexacoordinated octahedral configuration.

**Figure 4:**
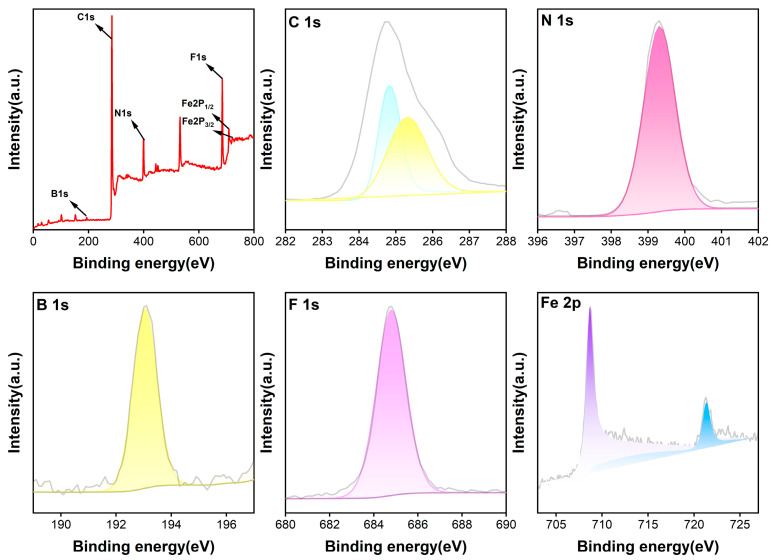
XPS spectra of the TPY-Ph-BPY-Fe(II) film.

The elemental states of the **TPY-Ph-BPY-Fe(II)** film were also studied using the X-ray photoelectron spectroscopy (XPS) technique. As shown in Figure 4, the full XPS spectra display distinct C 1s, N 1s, Fe 2p, B 1s, and F 1s peaks, aligning well with the metal ion and ligand precursors employed in the film assembly process. The Fe 2p spectrum reveals two characteristic peaks at 708.8 eV and 721.4 eV, which are, respectively, assigned to the Fe(II) 2p_3/2_ and 2p_1/2_ states [30]. No peaks associated with Fe(III) were detected, suggesting that Fe(II) exhibits significant chemical stability in the thin film. The B 1s and F 1s spectra exhibit two single-line peaks at 193.7 eV and 685.4 eV, which are attributed to the presence of a BF_4_^−^ counter-anion [25]. The N 1s spectrum shows a single-line peak at 399.9 eV, which is different from that of the pyridine-N peak around 398.7 eV [28], indicating the coordination effect between the TPY and BPY units of the asymmetric ligand and the Fe(II) ion. All of the above observations further prove the formation of the proposal 2D structure (Figure 2b) through the Fe^2+^-induced interfacial self-assembly of the asymmetric **TPY-Ph-BPY** ligand.

### 2.4. Electrochemical Performance of the **TPY-Ph-BPY-Fe(II)** Film

Cyclic voltammetry (CV) measurements of the as-prepared **TPY-Ph-BPY-Fe(II)** films were conducted within a three-electrode system in 0.1 M *^n^*Bu_4_NClO_4_/CH_3_CN electrolyte solution using the film-deposited ITO glass as the working electrode, a platinum wire counter electrode, and a silver wire-based reference electrode. As shown in Figure S6a, the film, prepared after the standing time of 3 h, exhibits a pair of reversible redox waves (Fe(II)/Fe(III)) with a half-wave potential (*E*_1/2_) of +1.33 V, a difference of the anodic (*E*_p,a_) and cathodic (*E*_p,c_) peak potentials (Δ*E*_p_) of 0.11 V, and a ratio of the anodic (*i*_p,a_) and cathodic (*i*_p,c_) peak currents (*i*_p,a_/*i*_p,c_) of 1.03 at a scan rate of 100 mV/s. To investigate the electron transfer mechanism of this film, CV tests were also performed by varying the scan rate range from 50 to 100 mV/s. The *i*_p,a_ and *i*_p,c_ were drawn in relation to the square root of the scan rate (*ν*^1/2^) (Figure S6b). The good linear relationships between them indicate that the redox behavior of the film is a diffusion-controlled process. Meanwhile, CV scans were also performed on the other films with different thicknesses at a scan rate of 100 mV·s^−1^. As shown in Figure S6c, the Δ*E*_p_ values increase with the film forming time, suggesting both enhanced film thickness and elevated charge transfer resistance. However, both *i*_p,a_ and *i*_p,c_ peak currents increase with the film forming time (Figure S6d), indicating the good conductivity of the obtained TPY-Ph-BPY-Fe(II) films.

### 2.5. Electrochromic Properties of the **TPY-Ph-BPY-Fe(II**) Film

The electrochromic characteristics of the **TPY-Ph-BPY-Fe(II**) film were evaluated using the spectroelectrochemical technique. The UV–vis spectrum changes were recorded under different applying potentials from 0 to +1.5 V in 0.1 M *^n^*Bu_4_NClO_4_/CH_3_CN electrolyte solution. As shown in Figure S7a, the film initially exhibits characteristic IL, LLCT, and MLCT absorption bands at λ_max,abs_ = 320, 372, and 573 nm, similar to those in the continuous titration process. As the increase of applied voltage moves from 0 to +1.5 V, both of the IL and LLCT absorption bands become intensified, while the MLCT absorption band is gradually diminished. Meanwhile, the film exhibits a clear color change from purple to light green (Figure S7b), which is relative to the metal core valence transformation from the Fe(II) state to the Fe(III) state. In addition, the electrochromic switching performances of the metal supramolecular film were also investigated. The transmittance changes at λ_max,abs_ = 573 nm were recorded using the double-step potentials between 0 V (neutral state) and +1.5 V (oxidized state) with an interval time of 30 s, as shown in Figure S7c. The optical contrast (Δ*T*%), which is defined as the difference in transmittance between the neutral state and oxidized state, was determined to be 39.5%. *t*_c_ and *t*_b_ were 2.3 s and 2.1 s (Figure S7d). The small difference between the coloring and bleaching time further indicates the good reversibility of the electrochromic film. The current efficiency (*CE*) was also calculated to be 426.2 cm^2^/C, which is much greater than those of the reported BPYs- or TPYs-Fe(II) based supramolecular systems (Table S2).

### 2.6. Electrochromic Performances of the Solid-State Device

**Figure 5:**
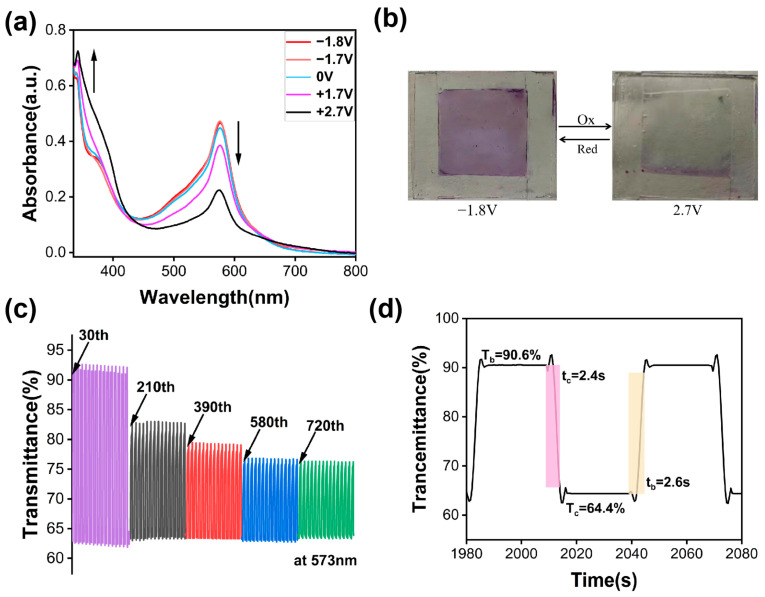
(**a**) Spectral variation of the solid-state device as applied potentials from −1.8 to +2.7 V. (**b**) The color changes of the devices at voltages of −1.8 V and +2.7 V. (**c**) Transmittance changes of the solid-state devices at λ_max, abs_ = 576 nm under applied voltages of −1.8 V and +2.7 V with an interval time of 30 s. (**d**) Calculation of the coloring and bleaching time of the solid-state device.

Solid-state devices were fabricated by employing the **TPY-Ph-BPY-Fe(II)** film as the electrochromic layer, and the UV–vis spectrum changes were recorded, as shown in Figure 5a. Compared with that of the film in the electrolyte solution, the characteristic ^1^MLCT absorption band is red-shifted to λ_max,abs_ = 576 nm. Meanwhile, due to the larger mass transfer resistance of the gel electrolyte [25], much higher redox potentials need to be applied to the solid-state device to maintain the complete oxidation and reduction states of the metal core. Upon increasing the applied potential to +2.7 V, the intensity of the characteristic ^1^MLCT transition peak is progressively decreased, resulting in a color turn from purple to light green (Figure 5b). Applying the reverse voltage of −1.8 V, the ^1^MLCT band and the film color are restored. The EC performances of Δ*T* % = 26.2% (Figure 5c), *t*_c_ = 2.4 s, *t*_b_ = 2.6 s (Figure 5d), and *CE* = 507.8 cm^2^/C were calculated. After continuous running of 900 cycles, the solid-state device still maintained 64.9% of the initial optical contrast, indicating its long-term cycling stability.

## 3. Experimental

### 3.1. Materials

All starting materials, including 4′-(4-boronatophenyl)-2,2′:6,2′′-terpyridine, 4-bromo-2,2′-bipyridine, polymethyl methacrylate (PMMA), propylene carbonate (PC), Pd(PPh_3_)_2_Cl_2_, K_2_CO_3_, and LiClO_4,_ were acquired from a commercial source and utilized directly without additional purification. The solvents employed for synthesis and characterization were of analytical purity. ITO-coated glass (CSG HOLDING Co., Ltd., Shenzhen, China; sheet resistance: 10 Ω/sq) was ultrasonically cleaned in acetone, ethanol, and ultra-pure water in turn, and dried under infrared light prior to use. The supporting electrolyte, *n*-tetrabutylammonium perchlorate (*^n^*Bu_4_NClO_4_), was prepared from *n*-tetrabutylammonium bromide and perchloric acid. The electrolyte was subjected to recrystallization twice from a hot pentanol/ethyl acetate solution, yielding colorless crystals, after which it was dried in a vacuum oven. (Warning! Perchlorate has the potential to explode and must be handled with care in small quantities).

### 3.2. Instrumentation

Nuclear magnetic resonance (NMR) spectra were recorded on a Bruker AV 400 spectrometer (Bruker, Billerica, MA, USA) with tetramethylsilane (Si(CH_3_)_4_) as an internal standard. Matrix-assisted laser desorption/ionization time-of-flight mass spectrometry (MALDI-TOF MS) was tested on an AB SCIEX-MALDI-TOF 5800 (AB SCIEX, Toronto, ON, Canada) using *trans*-2-[3-(4-*t*-butylphenyl-2-methyl-2-propenylidene]malononitrile as a matrix. Fourier transform infrared (FT-IR) spectroscopy was conducted on a Shimadzu IRTracer100 spectrometer (Shimadzu, Kyoto, Japan) with KBr pressed sheets for background acquisition. UV–vis–NIR spectra were obtained using a UH4150 spectrophotometer (Hitachi, Tokyo, Japan). Cyclic voltammetry (CV) was carried out on a CHI660A (CH Instruments Co., Shanghai, China) electrochemical workstation. A three-electrode system was used, consisting of ITO-coated glass serving as the working electrode, a silver wire as the reference electrode, and a platinum wire as the counter electrode. The measurements were performed in acetonitrile solution using 0.1 M *^n^*Bu_4_NClO_4_ as the supporting electrolyte. Field emission scanning electron microscopy (ZEISS Sigma500 FE-SEM, Zeiss, Jena, Germany) was used to examine the surface morphology of the film on the ITO electrode. An X-ray photoelectron spectrometer from Thermo Scientific K-Alpha, Waltham, MA, USA, was used to conduct tests under Al Kα radiation to characterize the elemental species, content, and valence information in the samples, and the binding energy resulting from the tests was corrected using the C 1s peak (284.8 eV).

### 3.3. Synthesis of the Target Ligand

Under an argon atmosphere, 4’-(4-boronatophenyl)-2,2′:6′,2′′-terpyridine (1.584 g, 4.5 mmol) was added to a three-necked flask and dissolved in toluene (50 mL). Then 4-bromo-2,2’-bipyridine (1.192 g, 5.1 mmol), Pd(PPh_3_)_2_Cl_2_ (0.063 g, 0.1 mmol), 8 mL of *t*-butanol, and 20 mL of 1 M potassium carbonate aqueous solution were added to the mixture in turn. The resulting light brown suspension was vigorously stirred and refluxed for 48 h. After cooling to room temperature, the mixture was directly filtered, and the filtrate was washed with water. The toluene phase was collected and concentrated to give solid precipitates. The crude products were dissolved in CHCl_3_ and then recrystallized from a CHCl_3_/MeOH mixture to give the final product (0.755 g, 47.7% yield). ^1^H NMR (400 MHz, CDCl_3_): *δ* 8.83 (s, 2H), 8.80–8.76 (m, 5H), 8.71 (d, *J* = 8.0 Hz, 2H), 8.52 (d, *J* = 8.0 Hz, 1H), 8.08 (d, *J* = 8.0 Hz, 2H), 7.96–7.87 (m, 5H), 7.65 (d, *J* = 8.0 Hz, 1H), 7.41–7.36 (m, 3H). ^13^C NMR (100 MHz, CDCl_3_): *δ* 156.5, 156.1, 156.0, 149.6, 149.5, 149.2, 149.1, 139.3, 138.8, 137.2, 137.1, 128.1, 127.8, 124.0, 121.7, 121.5, 119.1, 118.9. MALDI-TOF MS: *m*/*z* = 464.0 ([M]^+^, C_31_H_21_N_5_ requires 464.2).

### 3.4. Preparation of the Supramolecular Film

Initially, the ITO conductive glass (0.7 cm × 4.0 cm or 2.0 cm × 3.0 cm) was positioned on the bottom of a 100 mL beaker with the conductive side oriented upwards. Subsequently, the 10^−3^ M **TPY-Ph-BPY** ligand in 10 mL CHCl_3_ solution was added to submerge the ITO glass. Finally, about 10 mL 10^−2^ M Fe(BF_4_)_2_·6H_2_O aqueous solution was added dropwise onto the organic layer to form a clear liquid–liquid interface. After standing for a certain time, a purple thin film was formed at the water–oil interface. The lower CHCl_3_ solution was carefully removed by a dropper, leading to the adhesion of the purple film onto the surface of the ITO glass. The upper layer of the aqueous Fe^2+^ solution was then discarded, and the supramolecular film-deposited ITO glasses were obtained. Followed by washing with CH_2_Cl_2_ and H_2_O, the air-dried ITO glass was sealed for further use.

### 3.5. Gel Electrolyte Preparation

A mixture comprising 0.3 g LiClO_4_, 0.7 g PMMA, 2 mL PC, and 7 mL CH_3_CN was stirred under an argon atmosphere at room temperature until a transparent and sticky solution was obtained.

### 3.6. Solid-State EC Device Fabrication

The sandwich-type test devices were fabricated using a coordination film-coated ITO glass (size: 2.0 cm × 3.0 cm) as the working electrode and a blank ITO glass of the same size as the counter electrode. The above-mentioned gel electrolyte was drop-coated onto the working electrode. Then, the working and counter electrodes were face-to-face stacked by clips using the double-sided tape with a thickness of 60 microns as a spacer. Before testing, the devices were put in a desiccator to enable complete gelation over a period of 24 h.

### 3.7. Electrochemical and Spectroelectrochemical Testing

Electrochemical measurements were performed utilizing a three-electrode configuration, in which the supramolecular film-coated ITO glass slide served as the working electrode, a platinum wire functioned as the counter electrode, and a silver wire was employed as the reference electrode. The electrolyte solution was a 0.1 M *^n^*Bu_4_NClO_4_ CH_3_CN solution. A quartz cuvette with the same three-electrode configuration was used for the spectroelectrochemical measurements. The spectral variations under different electrochemical operational methods were recorded by a Hitachi UH4150 UV-Vis-NIR spectrophotometer coupled with a CHI660A electrochemical workstation, where a blank ITO glass was used as the reference. In addition, the spectroelectrochemical measurements of the solid-state ECDs were also carried out using a blank ECD without the electrochromic layer as the reference.

## 4. Conclusions

In this paper, an asymmetric two-arm polypyridine ligand **TPY-Ph-BPY** was successfully synthesized via the one-step Suzuki reaction. The **TPY-Ph-BPY-Fe(II)** supramolecular film was readily obtained using the Fe^2+^-induced liquid–liquid interfacial self-assembly process and easily transferred to a silicon wafer or ITO glass. This integrated method of online synthesis and ordered assembly of the 2D film facilitates the preparation and characterization of optoelectronic devices. The morphological features and elemental composition of the film were confirmed by the FT-IR, SEM-EDS, and XPS surface analysis techniques. The electrochemical analyses showed that the coordination polymer film had a diffusion-controlled and reversible Fe(II)/Fe(III)-based redox process. Furthermore, the film displayed reversible electrochromic behavior with a clear color change from purple to light green. Its solid-state device showed better electrochromic performances than other reported three-arm TPYs- and BPYs-based supramolecular systems, especially the much higher current efficiency (*CE*), more balanced electron transfer process, and longer cycling stability. This research provides a novel strategy for creating advanced two-dimensional nanomaterials with superior opto-electrochemical characteristics.

## Data Availability

The data presented in this study are available on request from the corresponding author.

## Supplementary Materials

The following supporting information can be downloaded at: https://www.mdpi.com/article/10.3390/molecules30061376/s1, Scheme S1: Synthetic route of the **TPY-Ph-BPY** ligand; Figure S1: 1H NMR of the **TPY-Ph-BPY** ligand; Figure S2: ^13^C NMR of the **TPY-Ph-BPY** ligand; Figure S3: The photo-pictures (**a**), SEM images (**b**), and the average film thickness versus the standing time (**c**) of the **TPY-Ph-BPY-Fe(II)** supramolecular films prepared after standing for 3 h, 6 h, 9 h, and 12 h; Figure S4: (**a**) Absorption spectra of the obtained films with different thicknesses; (**b**) variation of absorbance *λ*_max_ = 579 nm with film deposition time; Figure S5: FT-IR spectra of the **TPY-Ph-BPY** ligand and the **TPY-Ph-BPY-Fe(II)** supramolecular film; Figure S6: (**a**) Cyclic voltammograms of the **TPY-Ph-BPY-Fe(II)** supramolecular film coated ITO electrode at different scan rates (50–100 mV/s). (**b**) The anodic (*i*_p,a_) and cathodic (*i*_p,c_) peak currents as a function of the square root of the scan rates; (**c**) Cyclic voltammetry (CV) curves of the obtained films with different thicknesses; (**d**) Variation of peak current with film deposition time; Figure S7: (**a**) UV–vis spectrum changes of the **TPY-Ph-BPY-Fe(II)** supramolecular film coated ITO electrode over a range of 0–1.5 V. (**b**) The color variation images of the **TPY-Ph-BPY-Fe(II)** supramolecular film at 0 V and +1.5 V. (**c**) Transmittance changes of the **TPY-Ph-BPY-Fe(II)** supramolecular film at λ_max,abs_ = 573 nm under the double-step voltages of 0 V and +1.5 V with an interval time of 30 s. (**d**) Calculation of the coloring time and bleaching time of the EC film; Table S1: Energy dispersive spectroscopy (EDS) data of the **TPY-Ph-BPY-Fe(II)** supramolecular film; Table S2: Electrochromic performance of the previously reported three-arm TPYs- and BPYs-based supramolecular systems and the asymmetric ligand-based supramolecular film in this work.
