# Supplementary material for: Novel Fe(II)-Based Supramolecular Film Prepared by Interfacial Self-Assembly of an Asymmetric Polypyridine Ligand and Its Electrochromic Performance
Source: Molecules. 2025 Mar 19;30(6):1376. doi: 10.3390/molecules30061376 (PMC11944750; doi:10.3390/molecules30061376)
Supplement: Supplementary file 1 [file molecules-30-01376-s001.zip › molecules-3500384-supplementary.pdf]

Supplementary Material

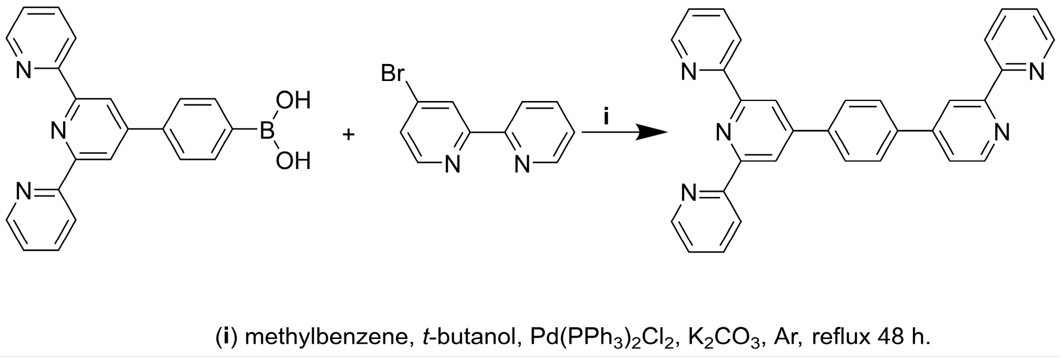

Scheme S1. Synthetic route of the **TPY-Ph-BPY** ligand.

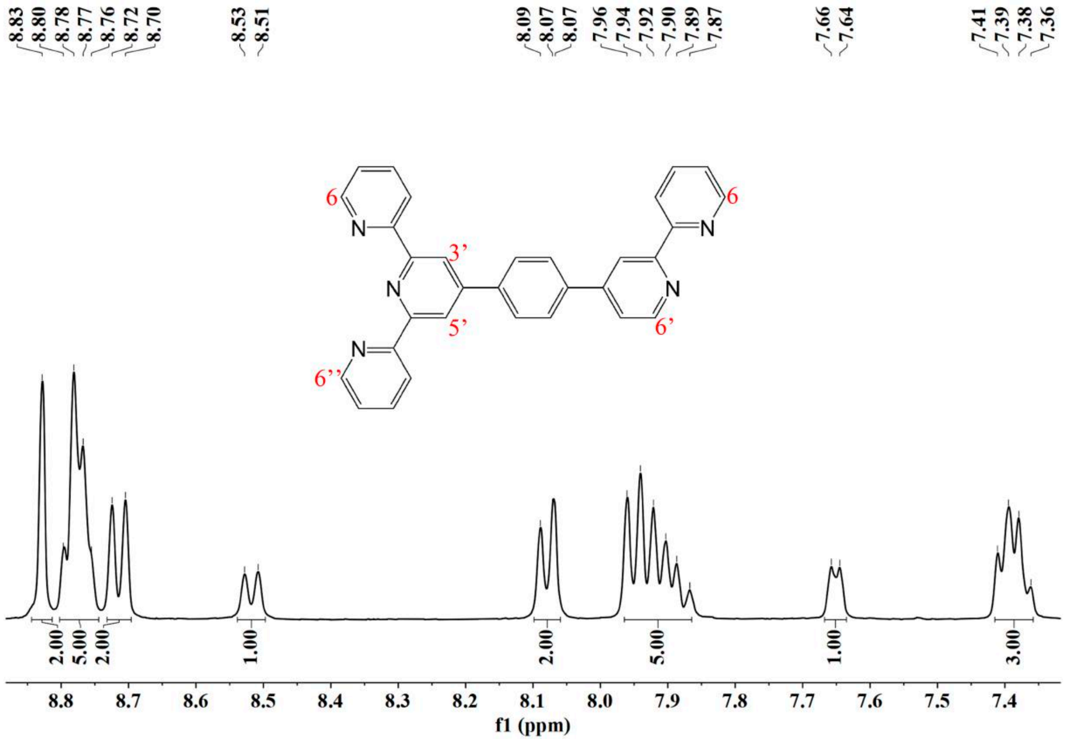

Figure S1 1H NMR of the **TPY-Ph-BPY** ligand.

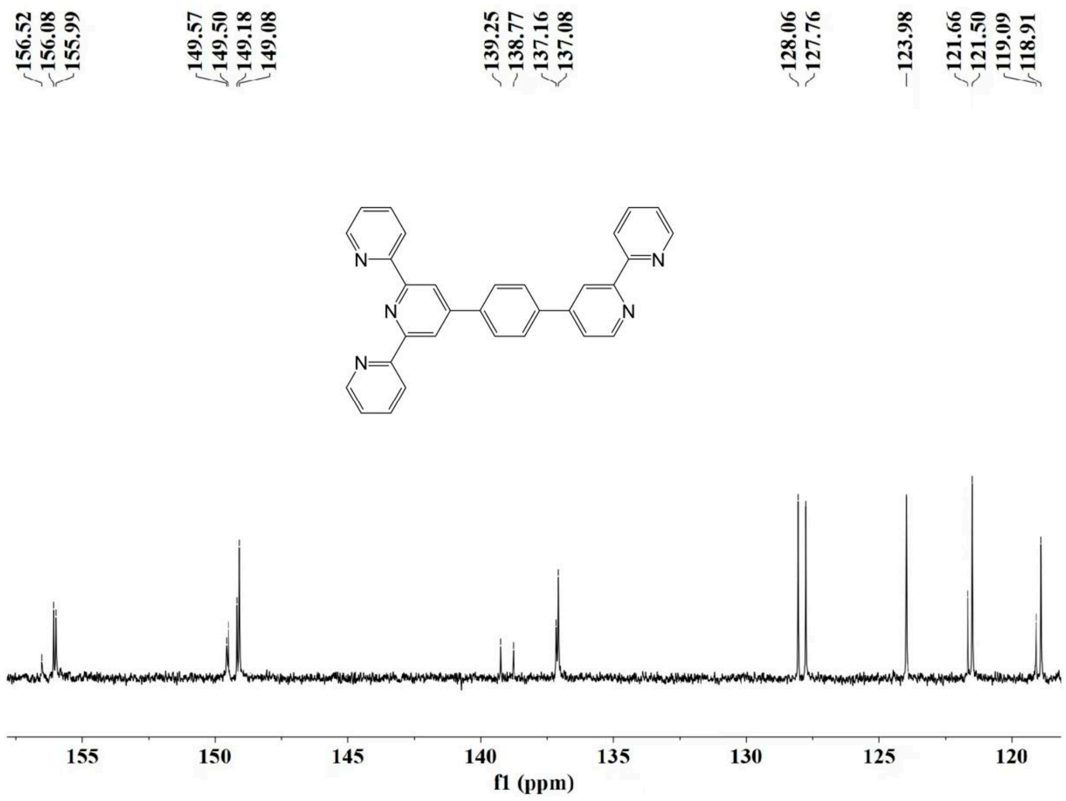

Figure S2 ^{13}C NMR of the **TPY-Ph-BPY** ligand.

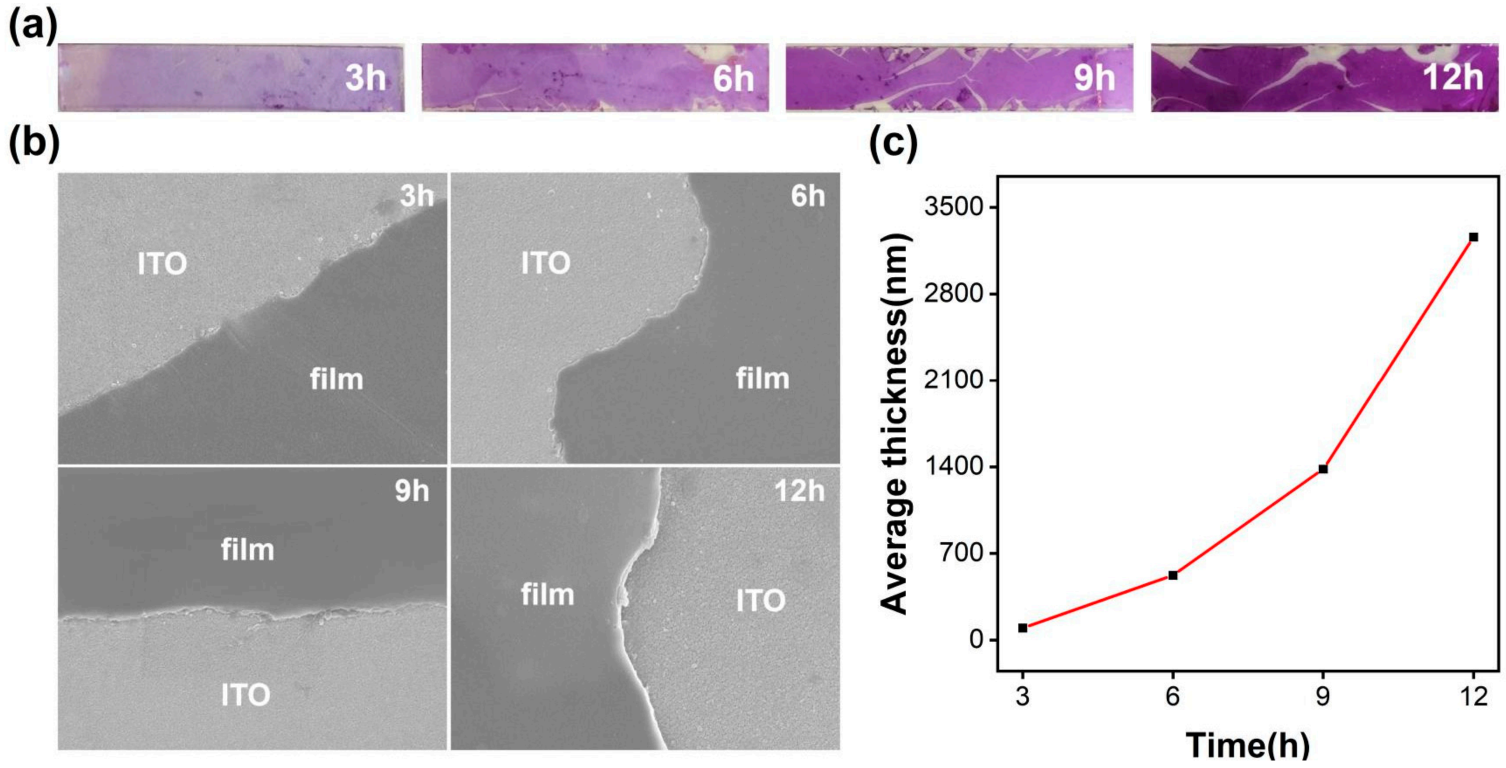

Figure S3 The photo-pictures (a), SEM images (b) and the average film thickness versus the standing time (c) of the **TPY-Ph-BPY-Fe(II)** supramolecular films prepared after standing for 3 h, 6 h, 9 h, and 12 h.

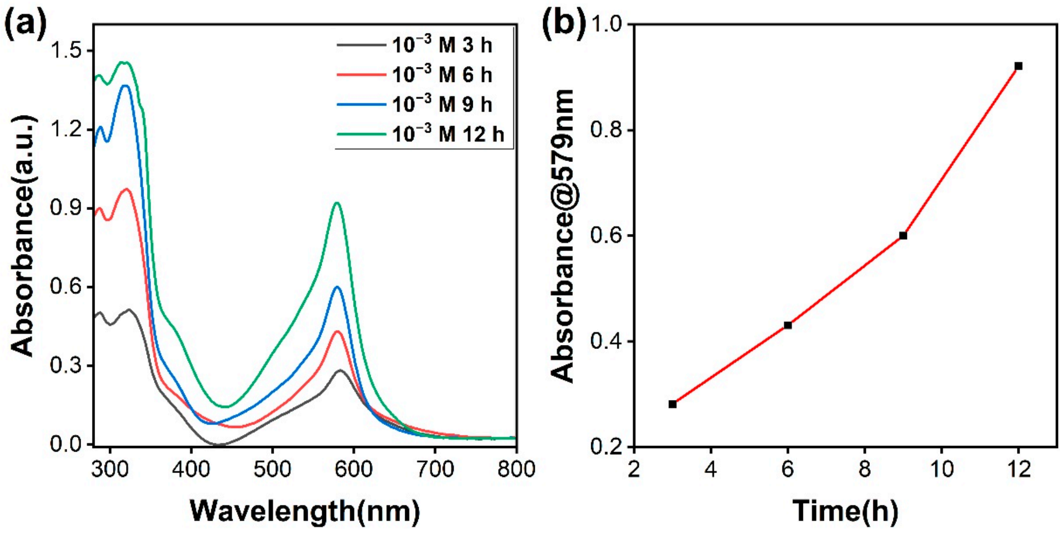

Figure S4 (a) Absorption spectra of the obtained films with different thicknesses; (b) Variation of absorbance λ_{max} = 579 nm with film deposition time.

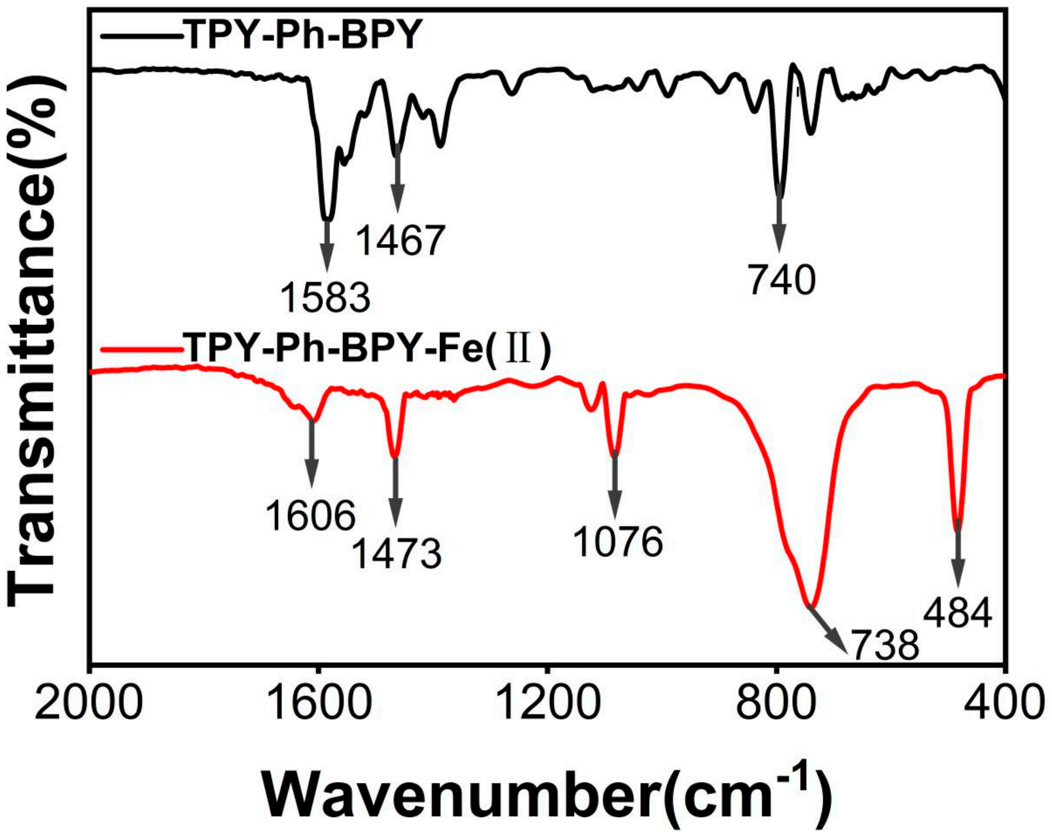

Figure S5 FT-IR spectra of the **TPY-Ph-BPY** ligand and the **TPY-Ph-BPY-Fe(II)** supramolecular film.

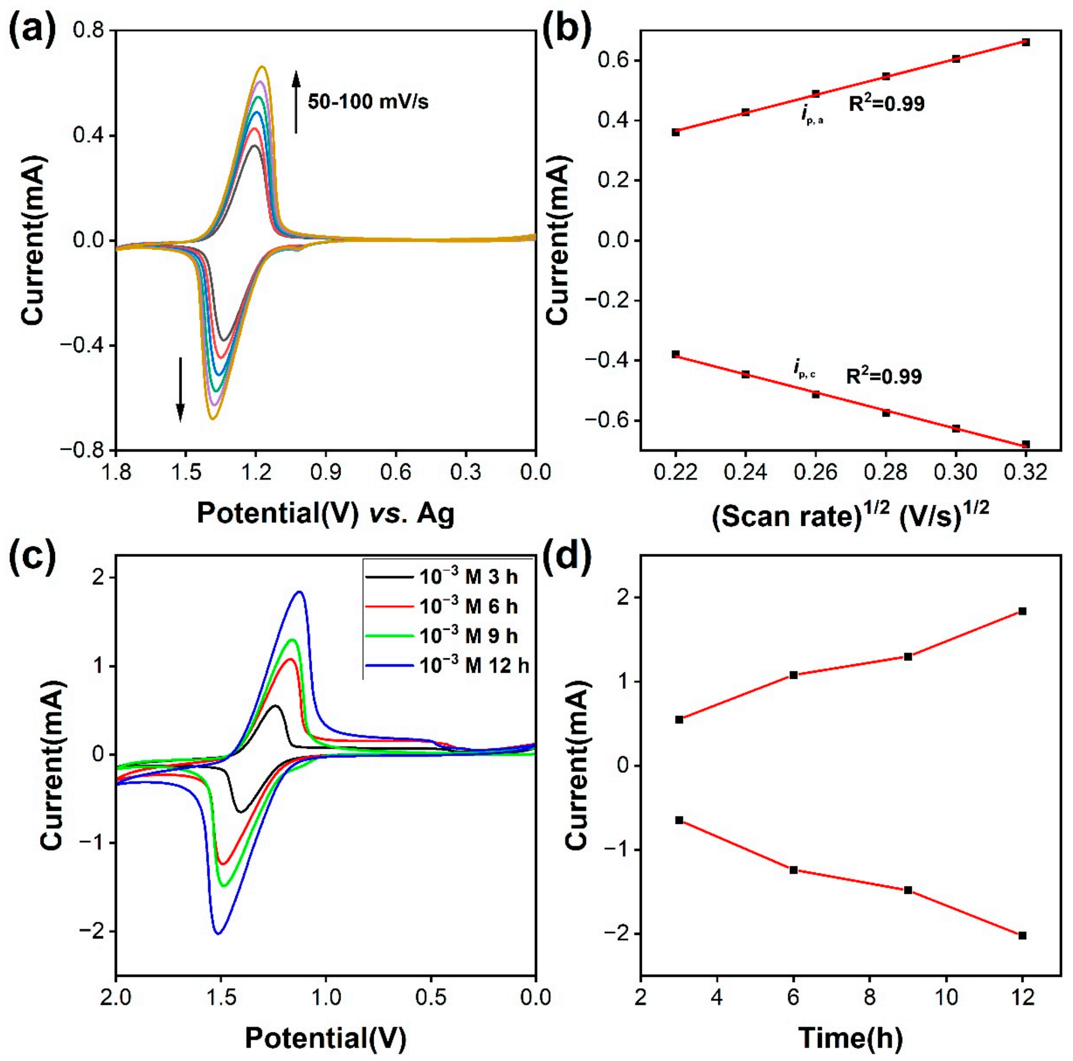

Figure S6(a) Cyclic voltammograms of the **TPY-Ph-BPY-Fe(II)** supramolecular film coated ITO electrode at different scan rates (50-100 mV/s). (b) The anodic ($i_{p,a}$) and cathodic ($i_{p,c}$) peak currents as a function of the square root of the scan rates; (c) Cyclic voltammetry (CV) curves of the obtained films with different thicknesses; (d) Variation of peak current with film forming time.

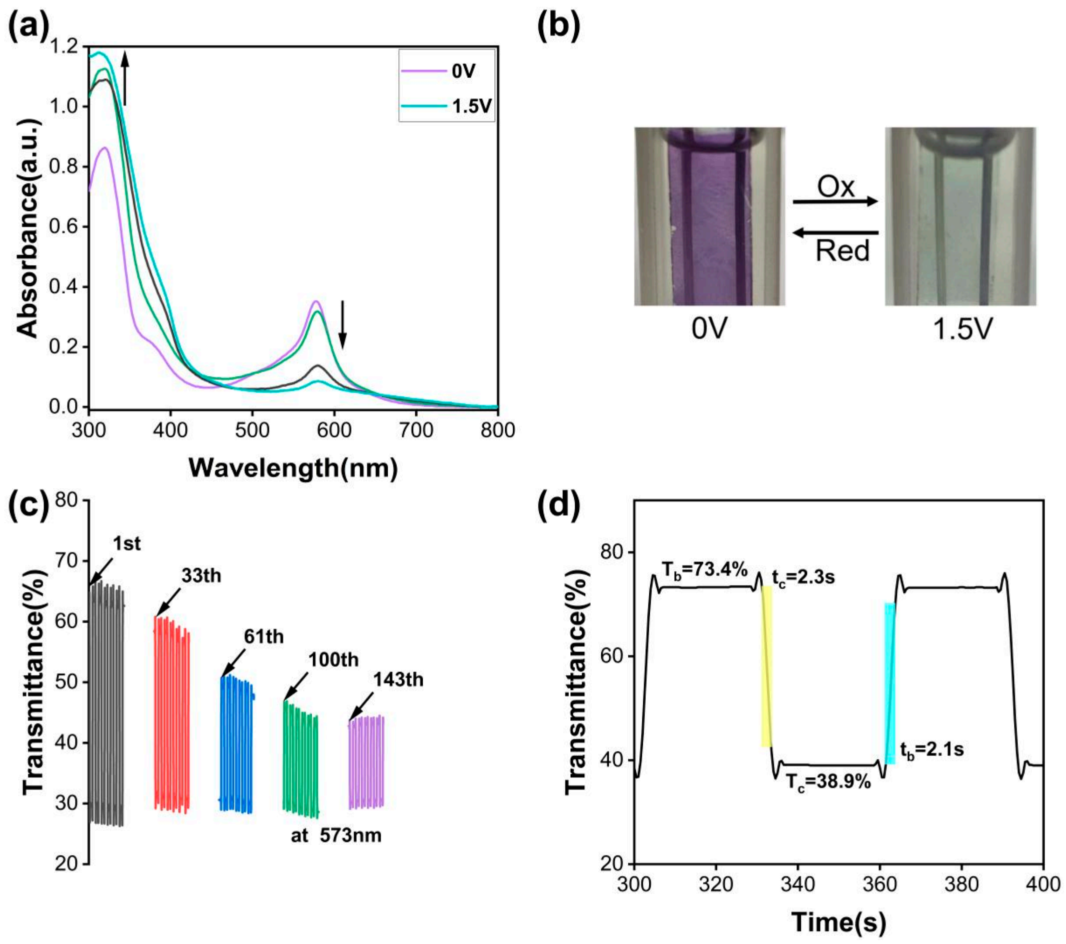

Figure S7 (a) UV-Vis spectrum changes of the **TPY-Ph-BPY-Fe(II)** supramolecular film coated ITO electrode over a range of 0-1.5 V. (b) The color variation images of the **TPY-Ph-BPY-Fe(II)** supramolecular film at 0 V and +1.5 V. (c) Transmittance changes of the **TPY-Ph-BPY-Fe(II)** supramolecular film at $\lambda_{max,\ abs}$ = 573 nm under the double-step voltages of 0 V and +1.5 V with an interval time of 30 s. (d) Calculation of the coloring time and bleaching time of the EC film.

Table S1. Energy dispersive spectroscopy (EDS) data of the **TPY-Ph-BPY-Fe(II)** supramolecular film.

ELEMENT	C	N	B	F	Fe
ATOMIC PERCENT(%)	65.75	6.98	11.78	14.33	1.16

Table S2. Electrochromic performance of the previously reported three-arm TPYs- and BPYs-based supramolecular systems and the asymmetric ligand based supramolecular film in this work.

LIGAND STRUCTURE	TYPE	ΔT(%)	t_c(s)	t_b(s)	CE(cm²/C)	STABILITY	λ_{max}(nm)	REFERENCE
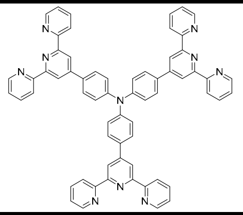	Liquid	22.3	0.5	0.4	141.7	500	580	[20]
	Solid	-	1	0.9	-			
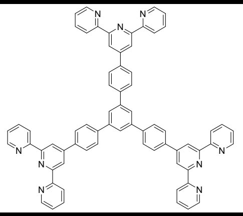	Liquid	50.7	0.2	0.4	383.4	800	580	[21]
	Solid	-	-	-	-			
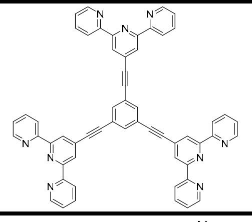	Liquid	-	-	-	-	1000	588	[22]
	Solid							
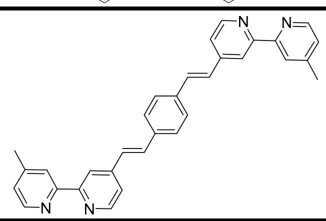	Liquid	57.0	0.5	0.5	382.0	1500	568	[24]
	Solid	61.0	1.7	2.5	-	300		
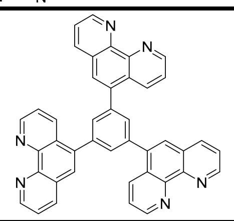	Liquid	55.0	2.9	3.3	230.0	15000	518	[23]
	Solid	65.0	6.0	9.0	-	-		
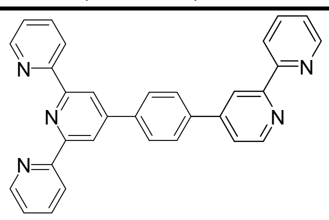	Liquid	39.5	2.3	2.1	426.2	150	573	This work
	Solid	26.2	2.4	2.6	507.8	900	576	
